# Bourbon Virus in Wild and Domestic Animals, Missouri, USA, 2012–2013

**DOI:** 10.3201/eid2509.181902

**Published:** 2019-09

**Authors:** Katelin C. Jackson, Thomas Gidlewski, J. Jeffrey Root, Angela M. Bosco-Lauth, R. Ryan Lash, Jessica R. Harmon, Aaron C. Brault, Nicholas A. Panella, William L. Nicholson, Nicholas Komar

**Affiliations:** Centers for Disease Control and Prevention, Fort Collins, Colorado, USA (K.C. Jackson, A.C. Brault, N.A. Panella, N. Komar);; US Department of Agriculture, Fort Collins (T. Gidlewski, J.J. Root);; Colorado State University, Fort Collins (A.M. Bosco-Lauth);; Centers for Disease Control and Prevention, Atlanta, Georgia, USA (R.R. Lash, J.R. Harmon, W.L. Nicholson)

**Keywords:** arbovirus, Bourbon virus, viruses, Thogotovirus, ecology, serologic analysis, neutralizing antibodies, plaque reduction neutralization test, vector-borne infections, zoonoses, Missouri, United States

## Abstract

Since its recent discovery, Bourbon virus has been isolated from a human and ticks. To assess exposure of potential vertebrate reservoirs, we assayed banked serum and plasma samples from wildlife and domestic animals in Missouri, USA, for Bourbon virus–neutralizing antibodies. We detected high seroprevalence in raccoons (50%) and white-tailed deer (86%).

Bourbon virus (BRBV) was first isolated from a febrile patient with a history of tick bites in Bourbon County, Kansas, USA; the patient later died from severe illness in 2014 (*1*). Several additional human BRBV infections were reported subsequently from the midwestern and southern United States (*2*). BRBV belongs to the family *Orthomyxoviridae*, genus *Thogotovirus*, which is distributed worldwide and includes Araguari, Aransas Bay, Dhori, Jos, Thogoto, and Upolu viruses (*1**,**3*). Thogoto and Dhori viruses have been associated with human disease (*4**–**6*). Viruses within the genus *Thogotovirus* have been associated with hard or soft ticks (*7*). Recent studies suggest that the lone star tick (*Amblyomma americanum*) is involved with BRBV transmission (*2**,**3**,**8*). These ticks feed primarily on mammals, which might play a role in BRBV ecology.

We evaluated banked animal serum and plasma for evidence of BRBV infection by using the plaque-reduction neutralization test (PRNT) to detect BRBV-reactive antibodies. We tested specimens of white-tailed deer (*Odocoileus virginianus*), raccoon (*Procyon lotor*), Virginia opossum (*Didelphis virginiana*), and various other mammals and birds from northwest Missouri, USA, for neutralizing antibodies against BRBV to identify naturally exposed host species and to implicate potential zoonotic amplifiers.

We collected specimens from wild and domestic vertebrates as described (*9*). We performed PRNTs on serum and plasma samples by using Vero cell culture as described (*9*). In brief, we initially screened samples by diluting them 1:5 and mixing them with an equal amount of BRBV suspension containing ≈100 PFUs/0.1 mL. Samples that showed >70% reduction of plaques were confirmed by serial 2-fold titration in duplicate from serum dilutions of 1:10–1:320. We considered 70% PRNT titers >10 as positive.

We screened serum and plasma samples from 301 birds and mammals for BRBV-neutralizing antibodies. A total of 48 (30.8%) of 156 mammalian serum samples were positive at the 70% neutralization level (Table). Mammals with evidence of past infection included domestic dogs, eastern cottontail, horse, raccoon, and white-tailed deer. None of 26 avian species were seropositive (Appendix Table).

**Table:** PRNT_70_ results for mammals tested for Bourbon virus–neutralizing antibodies, Missouri, USA, 2012–2013*

Common name	Species name	No. positive/no. tested	Titer	Proportion positive (95% CI)
Domestic cat	*Felis catus*	0/2	<10	0 (0–0.66)
Domestic dog	*Canis lupus familiaris*	2/13	10–>320	0.15 (0.04–0.42)
Eastern cottontail	*Sylvilagus floridanus*	2/9	>320	0.22 (0.06–0.55)
Fox squirrel	*Sciurus niger*	0/4	<10	0 (0.0–0.49)
Horse	*Equus caballus*	1/24	20	0.04 (0.007–0.20)
Raccoon	*Procyon lotor*	31/62	10–>320	0.50 (0.38–0.62)
Virginia opossum	*Didelphis virginiana*	0/28	<10	0 (0.0–0.12)
White-tailed deer	*Odocoileus virginianus*	12/14	10–>320	0.86 (0.60–0.96)

*PRNT_70_, 70% plaque reduction neutralization titer.

BRBV is probably transmitted to humans and other vertebrates by the lone star tick, an abundant arthropod in the south-central United States (*8*). This virus was cultured from these ticks in northwestern Missouri in 2013 and eastern Kansas in 2015 (*2*,*8*). Our results indicated that mammals are frequently exposed to BRBV. This finding was expected because lone star ticks feed primarily on mammals, and rarely on birds. Our study corroborates that birds are not involved in BRBV transmission, and our data establish that the vertebrate host range for infection now includes >5 mammalian species, 2 of which are domestic animals (dogs and horses). Of the wildlife species, the seropositivity rate for white-tailed deer was high (86%), whereas Virginia opossums, despite a moderate sample size (n = 28), showed no evidence of virus exposure. Deer and raccoons (seroprevalence 50%) could be useful wildlife sentinels for tracking the geographic distribution of BRBV. Dogs (seroprevalence 15%) and horses (seroprevalence 4%) merit further consideration among domestic animals for use as sentinels for either tracking virus activity or as an early warning system for mitigation of human risk. Because of limited sampling, we observed no statistically significant difference in seroprevalence between these 2 species.

A limitation of our study was small sample sizes, which reduces the accuracy of the seroprevalence measurements. Furthermore, serologic data provide indirect evidence of virus infection, rather than the detection of the virus itself or its parts (i.e., antigen or nucleic acid). However, a closely related congener could exist and generate cross-reactive antibodies to BRBV, causing false-positive results in our assay. Nevertheless, the PRNT is generally considered the standard for serologic assays.

In conclusion, we have demonstrated that nonhuman vertebrates are exposed to BRBV. These findings are useful for future public health efforts and to better understand the ecology of BRBV. Specifically, we identified 2 candidate wildlife sentinels and potential domestic sentinels for tracking and possible early warning of BRBV transmission risk. However, whether any of these mammalian species are competent amplifier hosts for BRBV remains to be determined.

Appendix Additional information on Bourbon virus in wild and domestic animals, Missouri, USA, 2012–2013.
