# Supplementary material for: Bourbon Virus in Wild and Domestic Animals, Missouri, USA, 2012–2013
Source: Emerg Infect Dis. 2019 Sep;25(9):1752–3. doi: 10.3201/eid2509.181902 (PMC6711231; doi:10.3201/eid2509.181902)
Supplement: Appendix — Additional information on Bourbon virus in wild and domestic animals, Missouri, USA, 2012–2013. [file 18-1902-Techapp-s1.pdf]

Article DOI: https://doi.org/10.3201/eid2510.181902

Bourbon Virus in Wild and Domestic Animals, Missouri, USA, 2012–2013

Appendix

Appendix Table. Bird species tested for antibodies to Bourbon virus, Missouri, USA, 2012–2013

Common name	Species name	No. positive/no. tested
American goldfinch	*Spinus tristis*	0/16
Baltimore oriole	*Icterus galbula*	0/2
Black-capped chickadee	*Poecile atricapillus*	0/13
Carolina wren	*Thryothorus ludovicianus*	0/4
Chipping sparrow	*Spizella passerina*	0/1
Common yellowthroat	*Geothlypis trichas*	0/1
Downy woodpecker	*Dryobates pubescens*	0/6
Eastern bluebird	*Sialia sialis*	0/1
Eastern towhee	*Pipilo erythrophthalmus*	0/1
Eastern wood-pewee	*Contopus virens*	0/5
Field sparrow	*Spizella pusilla*	0/1
Gray catbird	*Dumetella carolinensis*	0/1
Hairy woodpecker	*Dryobates villosus*	0/3
House sparrow	*Passer domesticus*	0/14
House wren	*Troglodytes aedon*	0/1
Indigo bunting	*Passerina cyanea*	0/7
Least flycatcher	*Empidonax minimus*	0/1
Mourning dove	*Zenaida macroura*	0/15
Northern cardinal	*Cardinalis*	0/24
Red-bellied woodpecker	*Melanerpes carolinus*	0/1
Red-eyed vireo	*Vireo olivaceus*	0/4
Rose-breasted grosbeak	*Pheucticus ludovicianus*	0/1
Summer tanager	*Piranga rubra*	0/4
Tufted titmouse	*Baeolophus bicolor*	0/7
White-breasted nuthatch	*Sitta carolinensis*	0/4
Wild turkey	*Gallopavo meleagris*	0/7
Total	26 species	0/145
